# Brief behaviour change counselling in non-communicable diseases in Mangochi, Southern Malawi: a hypothetical acceptability study

**DOI:** 10.1186/s40814-022-01032-0

**Published:** 2022-03-24

**Authors:** Prosper Lutala, Adamson Muula

**Affiliations:** 1 School of Public Health & Family Medicine, College of Medicine, University of Malawi, Zomba, Malawi; 2 NCDs-Brite Consortium, College of Medicine, University of Malawi, Zomba, Malawi; 3 Africa Centre of Excellence in Public Health and Herbal Medicine (ACEPHEM), Zomba, Malawi; 4 College of Medicine Mangochi Campus, P.O. Box 431, Mangochi, Malawi

**Keywords:** Brief behaviour change counselling, 5 As, Motivational interviewing, Hypertension, Diabetes, Mangochi, Southern Malawi, Qualitative study

## Abstract

**Introduction:**

Brief behaviour change counselling (BBCC) approaches have shown some potential in reversing and/or decreasing the effects of behavioural risk factors (harmful alcohol, smoking, physical inactivity and unhealthy diets). However, BBCC is new in Malawi. Thus, we explored the acceptability of brief behaviour change counselling using 5 As and a guiding style from motivational interviewing (BBCC + 5 As + GS) among health providers, policy-makers and patients living with noncommunicable diseases (NCDs) in the Mangochi district located in Malawi.

**Methods:**

An exploratory qualitative cross-sectional study used purposive sampling to select 44 respondents. We conducted group discussions with five focus groups that included patients. We also carried out nine key-informant interviews with healthcare providers and policy-makers. Data were managed and organized with Atlas.ti. cloud and analysed using the thematic framework approach.

**Findings:**

Several themes, categories, and their subcategories emerged from the interviews. Participants perceived the introduction and delivery of BBCC + 5As + GS in Mangochi as smooth. However, they predicted a few challenges such as time and space to conduct the intervention, cultural bottlenecks caused by low education level, age differences between healthcare providers and patients, low provider- to- patient ratio and high provider turnover. For this method to be adopted, a simplified format is deemed necessary to improve effectiveness with patients. This technique can only be sustained if training opportunities are provided and if positive testimonies are given by beneficiaries. Incorporation of a continuous quality improvement cycle targeting challenges must be part of the intervention. Participants perceived that BBCC will contribute to developing the listening ability of healthcare providers. This would help in providing personalized and cost-effective care relevant to Mangochi. The participants also perceived that BBCC + 5As + GS will be affordable, credible and useful.

**Conclusion:**

We found a high acceptability rate of BBCC among stakeholders in NCDs clinics in Mangochi. There are a number of areas where BBCC + 5As + GS could be improved to increase the acceptability. Accordingly, a study of feasibility and preliminary efficacy is warranted to determine other prerequisites for the implementation of a large-scale trial using this BBCC+ 5 As + GS, and to fully understand the implementation requirements of a full trial in Mangochi.

## Background

Diabetes and hypertension are currently the top causes of morbidity and mortality in Sub-Saharan Africa [1, 2]. More than a decade ago, projections showed that by 2030, 18 million people in Sub-Saharan Africa would have diabetes [3]. Based on most recent estimates, global epidemiological data indicate that the prevalence of diabetes in adults (18–99 years old) is projected to increase from 451 million people (8.4%) in 2017 to 693 million people (9.9%) in 2045 [4].

Through a national stepwise approach to studying noncommunicable diseases (NCDs), Malawi demonstrated that NCDs, such as diabetes, cardiovascular disease, cancers and chronic respiratory disease were already a burden on the healthcare system [5]. A more recent systematic review conducted in Malawi confirmed these findings and showed wide ranges for prevalence rates of diabetes (between 2.4 and 5.6%) and hypertension (between and 15.8 and 32.9%) [6], and high rates of the behavioural risk factors described elsewhere [7].

All chronic diseases, including diabetes and hypertension, share in common the presence of one or more underlying behavioural risk factors such as smoking, harmful alcohol use, unhealthy diet and physical inactivity [8]. Those behavioural risk factors play a critical role in disease genesis [9], modification and the onset of complications [8, 10]. However, these factors can also be managed using simple methods such as counselling [11, 12].

To fight recent increases in NCD prevalence rates, the Malawi government has developed and promoted curative, preventive, health promotional, systems-strengthening and policy measures [13]. Implementation of the above measures notwithstanding, psychosocial management of diabetes and hypertension in Malawi has only been partially addressed through routine group counselling for patients. However, unless paired with motivational interviewing, evidence supporting the effectiveness of group counselling in reducing behavioural risk factors remain weak, thus calling into question its effect on lifestyle behaviour change [1, 10, 14]. A brief intervention, considered as an alternative to group counselling, is defined as ‘a time-limited, patient-centred counselling strategy that focuses on changing patient behaviour and increasing patient compliance to therapy’ [13].

However, stand-alone brief behaviour change counselling (BBCC) is a provider-dominant, directive approach that confronts a passive recipient. Thus, it does not support internal change in patients, thereby reducing its ability to impact behavioural risk factors for chronic conditions and lifestyle behaviour [15]. Effective behaviour change requires a shift in the traditional roles of the provider and patient in the clinical encounter: from a ‘dominant, directing role’ of the provider, telling the patient what they should do and how they should do it, and from ‘passive recipient of… expert knowledge’ on the part of the patient [2]. In effective behaviour change, the provider becomes a facilitator of change in a supportive way, and an elucidator of self-motivation statements, and the patient becomes an expert in his own life. This shift increases motivation to change and minimises resistance to change [2].

However, this shift can only be achieved if the provider works within the spirit of motivational interviewing (MI) and uses a guiding style (GS) from MI. MI is a technique used to overcome resistance due to ambivalence towards change and lack of compliance with advice regarding behavioural risk factors by stimulating intrinsic motivation, eliciting the arguments for change from the patients or clients themselves and by helping them articulate and resolve their feelings of ambivalence. This requires an empathetic therapeutic style focused on active listening to understand patients’ perspectives and experiences [14]. GS is an approach that applies three principles of MI: ‘engage with and work in collaboration with the patient/client; emphasize their autonomy over decision making; and elicit motivation for change from the patient, rather than trying to instil this in them’ [9]. These principles are achieved through a combination of the following skills: using open-ended questions, listening to the patient’s perspective by using brief summaries as well as reflective listening, informing after asking for permission and asking the patient to interpret what the information means to them. Using GS helps the provider to move from the role of a director to that of a guide in counselling. Furthermore, it helps the client/patient to understand why and how change is being encouraged. It also conveys empathy, breaks resistance to change, and elicits motivation from patients [16].

Despite reported positive effects of brief intervention using 5 As and a GS (BBCC+ 5As + GS) in preventing and controlling NCDs in clinical practice [8], many primary care providers are not knowledgeable enough to deliver this intervention effectively [8]. This is due to several barriers, such as lack of formal training in counselling [12], time constraints, language barriers, lack of patient adherence to change, poor knowledge of lifestyle modification and poor continuity of care [12, 13]. There are also some local factors which can facilitate or impede the impact of these barriers locally in Mangochi, Malawi.

Yao is the main local language in Mangochi district, though the national language, Chichewa, is used just as often by people who are more likely to be from outside the district, more educated or living in the main town and other larger trading centres. Several Christian denominations exist in the district, but Mangochi is known as the most predominantly Muslim district in Malawi, especially among its rural areas. Many patients have a low literacy level. They rely on small scale business, fishing or traveling to South Africa for temporary work. Furthermore, gender imbalance is typical to African and Muslim cultures, with women having less power than their male counterparts. This submissive position affects household decision-making processes, including those related to healthcare. The youth are also subordinate to adults, whose orders they are supposed to obey without any protest.

The present study will generate evidence on the acceptability of BBCC + 5As + GS that will complement elements of a feasibility study and be used in larger trials in the future. Several previous studies used BBCC to addressed behavioural risk factors [8, 17–21]. Their results suggested that in order to change behaviour, one needs to combine 5 A’s and motivational interviewing [8]. Moreover, Stead et al. [21] affirmed that the combination of pharmacotherapy and behavioural approaches was essential for such change. Lastly, the increase in capacity of primary healthcare led to closing knowledge gaps regarding brief behaviour change and led to a significant improvement in the adoption of the GS and completion of the steps of 5 As during BBCC + 5As + GS [17] in clinical practice.

Research on using brief interventions to affect lifestyle behaviour change has a long tradition in Western countries [22]. However, this field is still very new in Africa [20, 21, 23, 24]. A quasi-experimental study conducted in Nigeria to assess the effects of using BBCC + 5As on hypertension control among patients in a hypertension clinic showed some positive changes in blood pressure in the experimental group compared to the controls [19]. Comparing a group receiving motivational interviewing (MI) to one receiving normal care or simple advice among a cohort of smokers, a meta-analysis study demonstrated significantly increased quit rates among patients in MI group compared to those in control group [20]. This study by Lindson-Hawley and al [20]. further showed a higher quit rate in those counselled by primary care physicians compared to those managed by a non-health professional trained in counselling.

Previous South African studies have focused on implementing [13, 25] and evaluating BBCC training [12, 17]. A situational analysis of primary healthcare providers on brief behaviour change highlighted several deficiencies. These include lack of confidence in BBCC and in current approaches to training, time constraints, lack of integration of training throughout the curriculum, training based more on theory rather than on modelling and practice and lack of both formative and summative assessments during BBCC training [13]. Implementation of trainings has also been limited by a lack of patient education materials, poor continuity of care and record keeping, conflicting lifestyle counselling messages and lack of a supportive system [13]. To respond to these limitations, a new training model based on 5 As and a GS was designed, resulting in an 8-h course combining theory, modelling and simulated practice with feedback targeting nurse practitioners and doctors in the primary care setting [25]. A study using a before-and-after design aimed to measure the effect of a training model for BBCC + 5As + GS from MI in clinical practice showed significant improvements in various aspects of BBCC. The training model led to observed improvements in adoption of the guiding style [score at baseline: 2.0 (2.0–2.6) and score in clinical practice: 3.0 (2.7–3.3) *p* < 0.001] and completion of the 5 A steps [score at baseline 1.26 (1.12–1.4), and in clinical practice 1.75 (1.61–1.89) *p* < 0.001] [17]. To our knowledge, little is known about behaviour change related to NCDs or application of motivational interviewing with a guiding style to affect change in behavioural risk factors in Malawi.

Considering this research gap, we conducted the present study with the aim of exploring the acceptability of BBCC using 5 As and a GS from MI in addressing lifestyle behaviour change among patients with diabetes or hypertension, healthcare providers, and policy-makers involved in NCDs clinics in the Mangochi District of Southern Malawi.

## Methods

### Study design

An exploratory cross-sectional qualitative study was conducted to explore the in-depth perspective of all NCD clinic stakeholders regarding BBCC using 5 As and a GS from MI in addressing lifestyle behaviour change, and the gains in acceptability of this approach [26]. Further, our study was a hypothetical and prospective product acceptability analysis, as it examines future users’ responses to a product (in this case, BBCC).

### Study setting and participants

This study was conducted at two sites in Mangochi District in Southeast Malawi (out of 42 facilities in the district): one, Mangochi District Hospital, in a suburban location, and another, Monkey Bay Community Hospital, in a rural trading centre [27]. The Mangochi district has a population of 1,017,070, of which almost 52% (528,876) are women. The rationale for choosing these two sites were twofold: (1) NCD care in Mangochi district is currently provided only by these two clinics, and (2) including both the suburban and rural was important for having a diverse group of participants.

### Sampling and selection of study participants

The target population for this study was comprised of patients with diabetes or hypertension receiving treatment at the NCD clinics at the Mangochi District Hospital or Monkey Bay Community Hospital, health care providers (nurses, clinical officers and medical doctors involved in routine management of the two conditions) and policy-makers (district health management team [‘DHMT’] members) who could affect policies related to diabetes and hypertension at the Mangochi District Health Office (DHO).

We used a non-probability purposive sampling, which was deemed appropriate for the study [28]. Patients were eligible to participate in the study if they were at least 18 years of age, fluent in Yao or Chichewa (local languages) and registered for diabetes or hypertension treatment at one of the two selected clinics for at least six months prior to the study. For healthcare professionals and policy-makers/managers, inclusion criteria were that they had to be either working in an NCD clinic or a member of the DHMT directly involved with NCDs. Finally, we considered willingness to partake for all participants. We excluded from this study any critically ill patients (such as patients suffering from stroke, malignant hypertension, any severe clinical form of diabetes, coma or severe associated comorbidities such as severe malaria or meningitis). A total of 45 participants were approached during recruitment, out of more than 1000 patients and professionals from both sites, due to our focus on gaining in-depth perspective rather than the generalization associated with quantitative studies. Two out of the five policy-makers declined our request (either because of a busy schedule or for personal reasons) and 43 participants agreed to be included in the study. Of the 43 participants left, nine were either policy makers (three) or health care providers (six). We conducted this acceptability study during the period between October 2019 and early March 2020.

### Recruitment and data collection

Recruitment took place in the waiting areas of NCD clinics, for which we received permission through a verbal invitation made by the clerk and a research nurse for patients attending the clinic. The principal investigator recruited healthcare providers and the policy-makers through direct contact. In both cases, those interested in participating were given an information sheet with an informed consent form attached written either in Chichewa or Yao (for patients) or English (for healthcare providers and policy-makers). The principal investigator followed up with policy-makers and healthcare providers via telephone the next day to receive verbal consent and agree on a date, time, and location of the interview. Patients were directly contacted the same day by either the clerk or nurse and led to a side room, where they were given further information and asked to provide signed informed consent (or assent for illiterate patients). Sociodemographic data was collected prior to the interview, and anthropometric measurements (height, weight, waist and hip circumferences) were collected by the clerk using standard methods.

Participants were initially asked to watch a 15-min YouTube video in either English for key-informant interviews (KIIs) or Chichewa for focus-group discussion (FGDs) on BBCC + 5As + GS entitled ‘Conversion for Change- The 5 As and Tobacco cessation’, with authorization from the Behavioural Health and Wellness Program from the School of Medicine, University of Colorado (USA). Participants watched the video individually, either in the office of the principal investigator or of some policy-makers (for KII), or in groups of six to eight participants alongside the research team in the conference rooms at both study sites (for FGDs).

Immediately after watching the video, participants asked questions about the video and answers were provided by either the principal investigator or the research nurse. Data collection was conducted by three trained research assistants (two clinical officers, one state registered nurse and a clerk) and the principal investigator. Data was collected using semi-structured interviews with one of the two interview guides (one for health professional and another for patients), framed around our study’s conceptual framework, as described in the table of constructs (Table 1).

**Table 1:** Thematic index [29] of the acceptability framework

1. Introduction or design of brief behaviours change counselling in Mangochi a. Scope of brief behaviour change counselling b. Legal, technical, and administrative procedures c. Social mobilization d. Pre-intervention requirements e. Challenges in launching brief behaviour change counselling	
2. Delivery of brief behaviour change counselling a. Perceived ease b. Feeling comfortable to carryout brief behaviour change counselling c. Facilitators of brief behaviour change counselling d. Barriers (or challenges) to brief behaviour change counselling	
3. Future of brief behaviour change counselling in Mangochi clinics a. Adoption methods of brief behaviour change counselling b. Perceived sustainability measures for brief behaviour change counselling c. Perceived areas for improvement	
4. Intrinsic values of brief behaviour change counselling in Mangochi a. Cost of brief behaviour change counselling b. Perceived credibility of brief behaviour change counselling c. Perceived usefulness of brief behaviour change counselling	

First, the key-informant interviews (KIIs) were conducted in English by the principal investigator (PL), either in his office or in a place agreed upon by the participant. We used open-ended questions to allow participants to elaborate in their own words on each point raised during the interview. Throughout the interview, reflection, paraphrases and summaries were used to stimulate participants to provide more information and refocus them on the acceptability aspects of BBCC + 5A’s + GS. At the end, a brief summary of what had been discussed was provided by the researcher, and the participants were given the opportunity to add, subtract or correct information according to their understanding. Participants were further asked to add any ideas they felt could have been discussed but were not captured by the interview guide.

The focus group discussions were conducted by a registered nurse assisted by two clinical officers (both holding a 3-year post-secondary school diploma in nursing or clinical medicine) who had conducted several surveys (some qualitative) through the local university. They completed an 8-h BBCC and MI course comprised of lectures, modelling and practical demonstrations conducted by a certified trainer with a PhD in Family Medicine from the University of Stellenbosch (South Africa). Focus-group discussions were conducted mostly in Chichewa, with Yao being used to emphasize points or express key ideas. There were five focus-groups discussions (2 in Monkey-Bay and 3 at the district hospital), each comprised of 6 to 8 participants of both genders, according to their conditions (diabetes, hypertension and diabetes-hypertension comorbidity). The interview followed a specific interview guide tailored to the patients. At the end, the interviewers asked participants to add any information they thought would have been relevant that the interview guide had missed.

All interviews in both scenarios were digitally recorded after receiving participant consent. The median duration of interviews was 36 min (range 31 min and 1 h 30 min). At the end of both KIIs and focus-group discussions, participants were compensated with 1500 Malawi Kwacha (exchange rate at that time: $2.00 USD) for taking part in the study.

### Reflexivity and trustworthiness of the data

The principal investigator (PL) is a medical doctor with a special interest in subjective aspects of care following completion of a master’s degree in Family Medicine. He has some experience in translating those subjective aspects into qualitative inquiries [28, 30], and has worked at the Mangochi District Hospital in the diabetes-hypertension clinic periodically, where he has developed therapeutic relationships with a number of patients. Furthermore, by working for years in the same hospital, he also has made strong acquaintance with the policy-makers and health care providers.

Thus, to ensure the trustworthiness of the data, we considered credibility, transferability, dependability and confirmability [27] using multiple criteria [31–33].

### Conceptual framework for the study

As stakeholders, understanding BBCC + 5As + GS was the main focus for our study and so we decided to adopt a social acceptability component, defined as ‘patients’ assessment of the acceptability, suitability, adequacy or effectiveness of care and treatment’ [34]. In short, acceptability, from an inductive point of view, is defined as ‘how well the program is received by participants’ [31].

The process led to the conceptual frame as presented in Table 1.

**Fig. 1:**
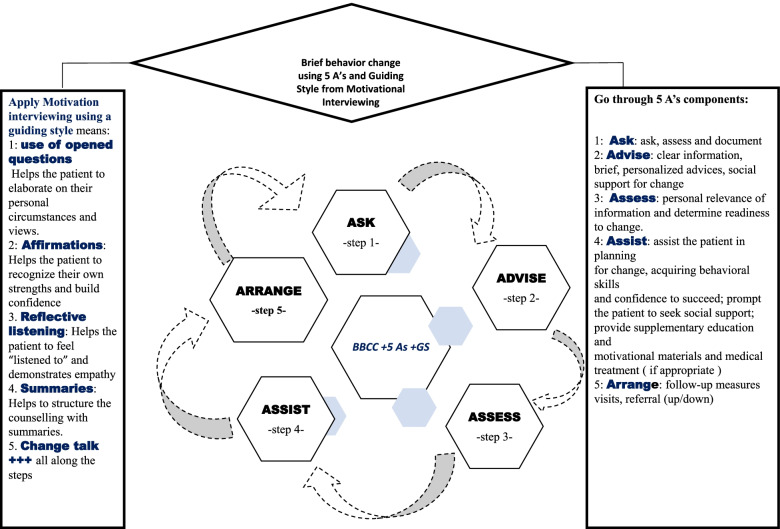
Brief behavior change counselling using 5 As and a guiding style from motivation interviewing (adapted from Everett-Murphy et al. [14]

The underpinnings of the conceptual framework in this study came from a model, a framework and several key concepts used in previous acceptability studies. We employed first the concepts of the technology acceptance model (TAM) [35, 36] used in adoption of information technology systems; and second, an acceptability and adoption framework used in result-based financing interventions [37]. The TAM constructs that were used in the present study were ‘ease of use’ and ‘usefulness’. In the result-based financing intervention framework, the used constructs included introduction and design of an intervention, acceptability of the intervention and sustainability of the intervention. Third, more acceptability constructs were added to our framework using an inductive approach from data: barriers and facilitators for BBCC, perceived areas for improvement and cost of BBCC (see Fig. 1). These constructs and concepts were used to design the interview guides which were used in both KIIs and focus group discussions. The guide and data analysis (deductive component) and the key constructs (usefulness, perceived ease of use and acceptability) in both models and constructs have been defined elsewhere [29, 38, 39].

### Data management and analysis

The digitally recorded data were transcribed verbatim by a research data clerk, who was a professional translator and Chewa native, fluent in Yao (grew-up in Mangochi), with 12 years’ experience in a Nutrition Research Unit within the Public Health Department of the College of Medicine, Mangochi Campus, University of Malawi. Transcripts were later translated into English before analysis using a theoretical framework approach [39].

The first author began analysis with familiarization of the text by reading and re-reading it several times as well as listening to the audiotape, modifying the transcript where doubts persisted. This was done in parallel with his colleague (who has a master’s in public health, a nursing background and teaches research methods at a college in Botswana). After familiarization, both researchers went line by line through the transcript to assign meaningful words or groups of words in a sentence. This code was either a piece of a sentence from the framework or an idea (deductive). A concept was anything that might be relevant to acceptability, from as many different perspectives as possible (inductive approach). This stage was conducted progressively using Atlas.ti cloud software. The researchers exchanged the two codebooks to compare the codes. Nine differences emerged, and through messages, the researchers brainstormed until a consensus was reached on the numbers and type of codes. The researchers then developed their analytic framework, which also expanded the study’s framework to capture the inductive elements which were not in the deductive component. Similar codes were grouped to give a category a thematic index [29, 40] as portrayed in Table 1.

Using Atlas.ti which contained the list of codes and categories, coding proceeded using those codes applied to the rest of remaining 43 transcripts as the analytical framework was applied. The text was then transferred to an Excel spreadsheet to enter the text into a matrix. The matrix included codes, categories and references to interesting or illustrative text from the transcripts.

Finally, reflection on the meaning of the data, similarities, differences, underlying meaning, etc. gave rise to memos which were used to introduce results during the write-up.

Atlas.ti Cloud software (Atlas scientific software development GmbH) [41] was used to organize data, present them in a manageable and systematic way and speed-up the analysis.

### Ethics approval and consent to participate

Ethical approval to conduct this study was obtained from the College of Medicine Research and Ethical Committee (COMREC No. P. 08/18/2454; October 24, 2018). Authorization to conduct this study was obtained from the Mangochi District Council through Mangochi District Health Research Committee prior to the submission to the local Institutional Review Board (IRB). All participants provided written or verbal informed consent if they were literate or illiterate, respectively, in accordance with the Declaration of Helsinki. We kept all information sources confidential by removing all participant’s identifiers and keeping data in a locked cabinet. We presented the quotes which illustrate the results after removing the participant’s identifiers using a code.

## Results/findings

### Sociodemographic characteristics of participants

In total, we conducted six KIIs with healthcare providers and three with policy-makers, as well as three focus group discussions with patients at Mangochi NCD clinics. Two policy-makers (out of five invited) and one provider declined to participate in the study. Three FGDs were conducted in Monkey Bay; one with a healthcare provider and two with patients. The mean participants’ age was 36 years old (range from 26 to 69 years); participants’ education levels were between standard 2 (second year of primary school) and an undergraduate degree (BSc.). Most of the patients were running small businesses. Three were civil servants, and the remaining were either housewives or smallholder farmers. The patients’ experience with diabetes or hypertension ranged from 18 months to 23 years.

### Themes identified

Four themes related to our research question were identified: design/introduction, delivery, intrinsic value and future of the intervention. Each theme had several subthemes that were divided in different categories. Below, we present major themes, with supporting quotes as applicable.

1. Introduction or design of brief behaviour change counselling

Two providers suggested that BBCC should be a cross-cutting intervention, introduced at all clinics within the district. A patient suggested implementation of BBCC + 5As + GS in the community to minimize workload and increase access to the intervention. Securing all authorizations, thorough sites assessment for readiness, large social mobilization, designing local BBCC guidelines to supplement existing ones developed in other countries and healthcare providers’ training were also considered key factors for BBCC + 5As + GS introduction.

… at the OPD (BBCC has to be introduced) as there are indeed a wide variety of conditions. We are talking of Sexual Transmission Infections like HIV, Syphilis, gonorrhoea even other non-communicable diseases like hypertension, diabetes, antenatal, even malignancies. (Healthcare provider, KII No. 7)

…while recognizing a lack of relevance of international BBCC’s guidelines to Malawi setup, I suggest, however their use during the implementation stage while waiting for the ones tailored to Malawi realities. (Healthcare provider, KII No. 1)

2. Delivery of brief behaviour change counselling

Participants perceived BBCC + 5As + GS to be easy and therefore felt they would be comfortable in administering it. However, they also identified some barriers which could impede its implementation if not addressed.

A. Perceived ease of brief behaviour change counselling

Two nurse managers said they saw BBCC as being easy based on their previous training and experiences. For patients, BBCC + 5 As + GS was based on the content of the advice they had previously been receiving during their routine follow-up visits to NCD clinics. For patients with history of behaviour risk factors, they perceived BBCC +5As + GS to be easy as it addressed some of the side-effects they have been experiencing. However, according to a provider, for BBCC + 5As + GS to be easy, enough time and good prior planning is needed.

(…) . Maybe the only issue will be in terms of time because counselling actually is not something that you do one time off; you need to have time and also you need to give time to the patients so as long as, maybe you are not so busy and you have really scheduled your counselling sessions (the intervention seems easy) (Management, KII No. 2)

It’s easy because (…) when we explain problems in our bodies [during current follow-up visits], they also ask what we eat and they tell us how we can protect our bodies by telling us the food we must eat or not because some of the diseases come from the food that we eat (Mangochi DHO Patient Focus Group 3, Patient No. 3)

B. Feeling comfortable to administer BBCC

Providers felt that, in general, they would likely be comfortable with administering BBCC + 5As + GS if they received appropriate training and hands-on experience, had enough time and an appropriate work space and developed a sense of teamwork with peer coaching in place to improve the intervention.

it’s an approach that is not very difficult to apply and, with appropriate training someone should be able to administer[it]….to add on top of that, it needs more practice, yes! … (healthcare provider KII No. 1)

I think staff can also be comfortable like what I said if we have time, but also space where to do the counselling... (Management KII No. 4)

III. Perceived facilitators of brief behaviour change counselling

Participants identified facilitators of BBCC, such as the inclusion of NCDs in government policy documents, recognition of NCDs as a public health problem, various trainings for prospective healthcare providers nationwide and deployment of specific monitoring and evaluation tools in districts across the country.

… the Government of Malawi already recognized the burden of NCDs, and it is included in the health sector strategic plans 2012-2017, 2018-2022. (…). Even the staff in the various public hospital… they have passed through one or two trainings to do with the NCDs…. But also on the other hand, there are good guidelines on the way these NCDs … [referring to Malawi treatment guidelines] have to be treated in these public hospitals, so this is also another third positive thing that can enable us to say we can settle the ground and start something about the intervention. (Healthcare provider KII No. 1)

IV. Perceived barriers to brief behaviour change counselling

We identified two levels of barriers to BBCC + 5As + GS, and they are as follows:

Lack of skills and empathy: interns, clinical officers and medical assistants (three to four years post-secondary training before certification) with little to no past exposure to NCDs, and little knowledge, attitude and skill, are overused in outpatient department. They lack necessary skills to run something like BBCC + 5As + GS. The highly qualified staff are working in the wards.

…and at the same time there is the issue of (I don’t know how I can put it) (…), but you know counselling is also a skill whereby whenever you are doing it someone has to put trust in you and you will be able to show that you are able to understand the person to whom you are giving the counselling… So, it has something to do with attitude or maybe personalities, but I don’t know how that can affect. (Management KII No. 3)

Rooms not conducive to privacy: The outpatient department in Mangochi is characterized by two rooms for external consultation to assist the many patients queuing outside. This leads to problems such as seeing more than one patient in the room at time, lack of chairs for some of the patients, very little time devoted to each patient, and a lack of privacy, etc.

…can be a problem [consultation rooms because this thing will need a place where there is privacy so that because during the consultation or the conversation, they might come up with things that are so sensitive… (KII no. 1, health care provider)

### Inadequate staffing level

Despite a high number of patients with NCDs in the clinic, there are no nurses or clinicians who work full-time at NCD clinics in Mangochi. The healthcare providers are on a monthly roster and work in NCD clinic based on their availability on their ward. Consequently, there is a high turnover of healthcare providers at the clinics, denying patients the opportunity to build the strong relationship with providers required for continuity of care. The scarcity of providers becomes an excuse for a few to unfortunately request payment for their service, in what is otherwise a free healthcare system.

Yeah, some barriers are going to be there, as you know in Malawian setting, we are always busy, we have few staff so may be the health workers will face the change as you always say when change wants to take place, it takes place little by little we need to discuss with them properly because we have a lot of work to be done, so may be the other healthy workers may say it’s going to be a lot of work, we are going to take a lot of time, (Management KII No. 3)

[...], if we have a Yao client speaking to another Yao health worker, that will smoothen up the process; unlike maybe, a Yao speaker and a Chewa who will be taken as an outsider, so the judgmental component…. (Care provider KII No. 5)

The other thing is even the education level of the people in this place can also have an impact to the outcome. (Care provider KII No. 1)

…however maybe the challenge could be …especially females… it is difficult [to state in public], ‘I do smoke (Policy-maker 1, KII No. 2)

3. Future of brief behaviour change counselling at NCD clinics in Mangochi

Over time, participants saw BBCC + 5As + GS as being either adopted or sustained, and suggested areas which may require some improvements.

A. Adoption of brief behaviour change counselling

Reports from some patients, managers and healthcare providers revealed that the BBCC was likely to be adopted in Mangochi because it is simple, clear and a cheaper way to address the root causes of NCDs, thus reducing disease burden, as shown in cases from previous patients and their behavioural risk factors.

Apparently, …. It’s [ simplified well] as long as you follow the steps outlined in the model ... (Management KII No. 2)

… if we have people on/to whom we have used these models and how effective it has worked (…) should have an evidence-based decision whereby we will dwell much on (…) how effective the model has been to these people. … [patients with successful stories can share their experience to others and attract more candidates] (Management KII No. 2)

Apparently, (…) yes, it can be adopted. otherwise, I think there are no limitations and I don’t see any problems actually in adopting the model. It’s well simplified as long as you follow the step outlined in the model (…), as long as you know exactly what to ask at each and every point of the step. Otherwise, I think it can really be adopted

I would love maybe, for the first three months if we have people to whom we have used this model and how effective it has worked to these people, so that we should have an evidence-based decision whereby we will dwell much on what we have done to these people and how effective the model has been to these people. So, when other districts are interested, we will show them that evidence that when we started these five A’s models we practiced to these clients or patients and out of these almost all of them managed to get the best (Management KII No. 2)”

B. Sustainability

Participants felt that for BBCC +5 As + GS to be sustained, attention should be paid to simple implementation, cheaper training models for providers, designing modes of payment (in for-pay facilities) which can protect the most vulnerable patients and putting in place a continuous quality assurance system to maintain high standards.

…continue to monitor how it is fairing and note some of the negatives [points for improvement] … ...” (Healthcare provider KII No. 1)

…, we need to orient the other health workers [in BBCC], maybe at the morning report [morning handover] or (…) if you are going to the health centres, [use that opportunity] we need to orient them [ those working in health centres] …. (Healthcare provider, KII No. 7)

### Perceived areas for improvement

Participants thought that it would be critical to increase the number of trained providers, including health surveillance assistants (HSAs or community health workers). This would help facilitate reaching people in remote areas, cut down on transportation costs and prevent patients from having to travel long distances only for BBCC. To minimize patient frustrations, participants suggested hosting separate days solely for counselling, outside the NCD clinic days for those who need it, consistency in medication availability, opening NCD clinics early, availability of providers fluent in both Yao and Chichewa and of providers with ages close to patients.

This approach can be good if there are doctors [ health workers irrespective of the cadre] especially for these (NCDs), not those ones that are committed to other work. (Patient, Focus group discussion 3 Participant 5)

Another one is assisting them in time, like opening the clinic at whenever they coming like at half past five just because there is congestion whenever they come (Healthcare provider, KII No. 8)

[...], if we have a Yao client speaking to another Yao health worker…, that will smoothen up the process; unlike maybe, a Yao speaker and a Chewa who will be taken as an outsider… so the judgmental component…. (Care provider KII No. 5)

The other thing is even the education level of the people in this place can also have an impact to the outcome. (Care provider KII No. 1)

…however maybe the challenge could be …especially females… it is difficult to [state in public] ‘I do smoke like young care provider to an old patient (Policy-maker 1, KII No. 2)

4. Intrinsic values of brief behaviour change counselling in Mangochi

We identified the acceptability of BBCC through its perceived internal value in three categories: perceived cost, credibility, and usability.

### Perceived cost of brief behaviour change counselling (in for-pay health institutions)

Participants had different opinions regarding what the cost of BBCC should be at for-pay facilities. They ranged from it being a free service, to a wide range of prices between 200 to 200,000 MWK). Other suggest the use of an existing pay system already available in Malawi, such as Service-Level Agreements used by government to cover costs incurred by less privileged individuals in remote areas for their maternal and infant care in mission hospitals.

I don’t think it can cost much, all that it needs is to have the staff present actually, who are able, who can be able to do the counselling using the model; (…) Healthcare Provider KII No. 2)

l…. already we have got what we call service level agreement in most of CHAM facilities. I think it [BBCC+ 5As + GS] can be within the service level agreement for sustainability in this case …. (Healthcare provider KII No. 2)

### Perceived credibility of brief behaviour change counselling

Participants perceived that BBCC fulfils the requirements for a good consultation in diverse ways. For example, BBCC would allow patients and providers to reach mutual understanding, avoid unnecessary shouting at patients, lead to change, have follow-up in which adjustments could be made, lead to patient satisfaction and improve quality of care.

…. The client feels relaxed and becomes so open to the doctor [ with BBCC + 5As + GS] …. (Patient, Focus group discussion 3, Participant No. 5)

…. this is a good approach because we, … we are very much disappointed, more especially when we have not been warmly welcomed and [blood] sugar levels rise [now] there and then, (…) (Patient, Focus group discussion 2 Participant No. 5)

### Perceived usefulness of brief behaviour change counselling

There was a general feeling that BBCC could lead to several outcomes as delivered by providers: provision of a diagnosis, having a high expectation of a good outcome for behavioural risk factors; responding to patients’ expectations; instituting correct treatment; motivating health-seeking behaviours; helping to provide detailed information; building trust; and ensuring continuity of care. Furthermore, it could decrease the number of patients seeking care by improving health, if providers adhere to the instructions provided though BBCC training.

...[unlike in the past], in this approach, the client will be able to explain all her or his problems without looking at how much time he or she has taken. (Patient, focus group discussion 3 Participant 2) (Patient, Focus group discussion 5 Participant No. 2)

(…) a good approach because everyone will be able to ask questions individually, not in a group. (…) to the doctor and tell him or her all problems (…) and the doctor will be able to answer to all problems of each and every individual (specifically). (Patient, Focus group discussion 3 Participant No. 2) (

**Fig. 2:**
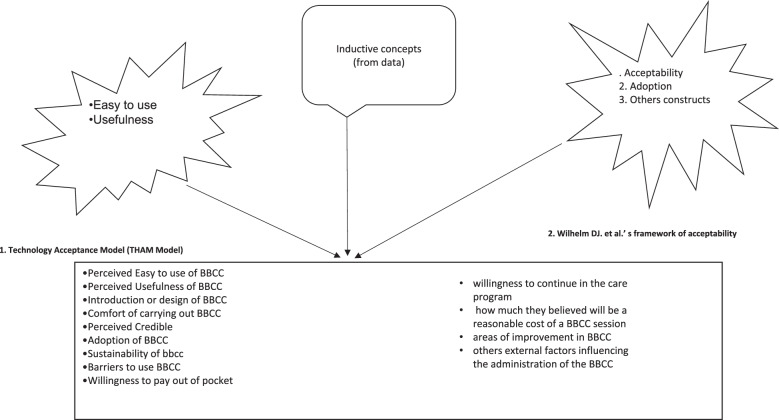
Study conceptual framework used to draw interview guide and in deductive/theory-driven analysis

Fig. 2)

## Discussion

To our knowledge, this is the first published study which explores the acceptability of BBCC + 5As + GS in Malawi among adults with hypertension or diabetes who receive treatment at NCD clinics. Although the study identified several themes (design, delivery both in the present and in the future, and perceived intrinsic value of BBCC among participants), the focus of our discussion will be on barriers/challenges in implementing BBCC + 5As + GS. They are worthy of consideration in an attempt to improve implementation of BBCC + 5 As + GS.

### Insufficient space for brief behaviour change counselling

Lack of appropriate space in terms of size (quite small), numbers (one space devoted to NCD clinics), lack of furniture (old chairs and tables, no examination couch) and lack of curtains or screens for counselling sessions were identified by a previous study as expected barriers to implementing BBCC [42]. This could potentially affect counselling sessions in several ways. First, it could compromise patient confidentiality and privacy (both auditory and visually), making patients feel unable to openly disclose sensitive issues to their healthcare providers. Evidence showed that lack of privacy due to inadequate infrastructure was a major barrier to implementing interventions dealing with sensitive issues [43, 44]. To address this weakness, and to bolster beside training and provision of basic equipment to healthcare facilities, BBCC implementation should support the infrastructure at the location where counselling sessions would be conducted. This will be done either through construction of new buildings or renovation of existing ones. Another solution can consist of having separate days for BBCC + 5As + GS sessions, uncoupled from drug refill visits, as suggested by participants. Furthermore, BBCC + 5As + GS should be rolled out in peripheral clinics and health centres to reduce congestion at current clinics and provide enough time to conduct the counselling sessions in a relaxed atmosphere. The closer the location of clinics to patients’ homes (with healthcare providers like community health worker from the same area as patients), the less patients will experience cultural barriers in terms of language, customs and practices.

### Human resource challenges

Participants identified several human resources challenges to the BBCC +5 As + GS in terms of staff numbers, workload, high turnover, high number of low/unskilled staff members, corruption and lack of allocation of designated staff to NCD clinics. Staff workload (and time limitations) was found among the main barriers to intervention implementation [45–47]. This high workload is due to staff shortage currently worsened by a well-known low doctor-patient ratio in Malawi estimated to be one doctor for every 18,000 people [48]. This low doctor-patient ratio extends to other cadres such as nurses, clinical officers and medical assistants, even community health workers (health surveillance assistants). Furthermore, this shortage would have a direct effect on BBCC implementation as the BBCC + 5 As + GS model as described in this study relies on one-to-one sessions, spending time with patients, and building strong relations and trust over time between healthcare provider and patient. To address this staff shortage, evidence-based solutions should be leveraged, such as the task-shifting used successfully in HIV and AIDS [49] and other NCD programming [50–52]. The level of cadre does not matter in BBCC + 5As + GS, as evidence has shown that BBCC can be carried out successfully by any staff, regardless of cadre [53]. Those who implement the intervention only need ensure that the work done by each cadre related to BBCC + 5As + GS aligns well with that cadre’s skills, competence and job-description. In the meantime, other well-tailored local innovative approaches that can alleviate this healthcare human resources issue, specifically as it pertains to BBCC + 5As + GS, should be explored.

Although using staff from others departments as a gap-filling measure in NCD management has been suggested before, most of the time such improvised staff, regardless of department of origin, are likely to feel ill-equipped as most have yet to undergo training in general NCDs [54]. The prior training requirement is important given the complex nature of BBCC + 5As + GS [43], the special populations at hand in these clinics (heavy drinkers, smokers, obese, etc.) and the specific conditions found in NCD’s clinics (diabetes, hypertension, etc.) [54]. Beyond its direct effects on human resources, high staff turnover can also affect the quality of care by decreasing the knowledge of the staff and reducing consistency in the implementation process [55]. Local mechanisms for staff retention, such as non-monetary incentives or monetary incentives with support from other well-funded programs, should be designed and implemented. However, as the direct outcome of incentives on high staff turnover in Mangochi is still unclear, there is a need to further research the impact of non-monetary versus monetary incentives (or incentives versus non-incentives) in retaining healthcare providers in NCD clinics in Mangochi.

### Lack of clinical practice guidelines

The lack of local NCDs clinical practice guidelines is a matter of extreme urgency in the healthcare system in Malawi. Used for recommendations in clinical practice or to set standards [56], clinical practice guidelines help improve healthcare quality, reduce variations in practice between healthcare providers, improve the morale of practitioners who have knowledge gaps and realign practice with theory and evidence [56]. As many healthcare providers working in these NCD clinics have received insufficient training, National NCD guidelines are needed to support a wide range of providers at both primary and secondary level of care in Malawi.

Beyond the issue of numbers, this shortage can further be placed in the context of the institutional culture of the Mangochi Health Districts. Patients raised the issue of providers not listening to them before prescribing or even shouting at them, requesting money to compensate the service offered, issues of workshops with attached allowances and absenteeism in some clinics (including NCDs). These behaviours can be sustained by the prevailing culture at a hospital or healthcare facility. This institutional culture issues leads to a significant misalignment of personal and organizational values [17] at the district hospital. Organizational values in institutional culture are currently characterized by ‘not sharing information’, ‘control and manipulation’, ‘blame’ and ‘power’ [57]. These values are far from the spirit of patient-centred care, which is at the heart of BBCC + 5As + GS. To realign institutional values to be closer to the spirit of patient-centred care, and thereby closer to BBCC+ 5As + GS, a previous study suggested instilling transformative leadership and making a concerted effort among stakeholders to turn the espoused values and culture into a lived reality [17]. In practical terms, there must be improvement in relationships, trust and communication among staff and management, and discussions about BBCC + 5As + GS with managers. Furthermore, it is necessary to ensure that organizational practices and processes are congruent with a patient-centred approach by prioritizing ongoing support for providers to offer BBCC + 5As + GS and wide implementation of BBCC through pilot programs in various clinics and others health centres [17].

Cultural barriers have to be understood in the context of the collective goals and aspirations of the group of people under consideration [58]. In this study, healthcare providers, managers and patients shared NCD care as a common concern, and sought the best management options for patients living with diabetes and/or hypertension in Mangochi. Our study reveals issues such as difficult intergenerational communication between healthcare providers and patients with wide age gaps, women having difficulties disclosing certain behavioural risk factors such as smoking or alcohol intake to providers or misunderstandings between healthcare providers and patients with low literacy levels. To consider how cultural beliefs influence these issues, we will define culture as ‘a collective identity, created by a summation of beliefs, behaviours and shared history and experiences of a group of people [59]’. In our case, based on anecdotal observations, we assume that participants in Mangochi are characterized by belonging to the Yao ethnic minority and are mostly Muslims, running small businesses, with generally low literacy levels, living in a matrilineal kinship system, with its specific gender imbalances characterized by females being considered powerless in society.

Referring to Hofstede and Hofstede’s [60] classification of a culture’s main features into dimensions that lead to better understanding of the way culture functions in a given society, we can say that people in Mangochi demonstrate features of four dimensions of this classification relevant to our analysis. We hypothesize these dimensions as alternative explanations for the position of females and youths relative to males and adults, respectively, in Mangochi. First, most of the Mangochi population are characterized by power distancing (i.e. ‘less powerful members of organizations and institutions accept and expect that power is distributed unequally’) [60], masculinity (i.e. ‘men are supposed to be assertive, tough and focused on material success; women are supposed to be more modest, tender and concerned with the equality of life’) [60], restriction (i.e. ‘gratification is not encouraged and is regulated by strict norms’) [60] and uncertainty avoidance (i.e. ‘The extent to which the members of a culture feel threatened by uncertain or unknown situations’) [60]. By considering these attributes as frameworks to contextualize and understand these cultural considerations, we can read meaning into each. The example of communication barriers between younger healthcare providers and senior patients may be explained by power distance and restriction. Both may find their sources in the Muslim and Yao cultures (and, by extension, are displayed by several other ethnic groups in sub-Saharan Africa). The traditional mode of living obliges younger people to abide by certain rules, often without a clear explanation.

Similarly, the superiority of men over women in Islam cannot be easily challenged, as it is socially accepted in Mangochi; it relates to the masculinity dimension of culture. The fact that women feel threatened in unknown situations may explain their hesitation to disclose to healthcare providers some behavioural risk factors that are not considered socially acceptable, such as smoking and drinking alcohol. Women fear stigmatization and social exclusion, even if they do not know how these will play out. The common misunderstanding between people who are literate (healthcare providers) and those who are illiterate (patients with no or low-level education) can be explained by uncertainty avoidance. In simple communication, even in the search for clarity from healthcare providers, patients misinterpret the message, leading to quarrels, mood changes and even ruptured relationships. Rather than scapegoating the perpetrators of behaviour, I intend for these interpretations to serve as generalizations of the facts (and thus a starting point for solutions) rather than to offer closure, conclusion or condemnation.

To address the cultural aspects of behaviour change, one needs to first place such change in the context of cultural dimensions either in favour of or against a particular behaviour. Although change in belief and behaviour are very difficult, putting them in context based on this classification (or others similar) can open up possibilities for interactions with concerned parties and maybe provide a path towards solutions. However, behaviour change takes time, and skilled and devoted healthcare providers are needed to conduct the necessary intervention.

### Strengths and limitations of this study

First, the strength of this study lies in the diversity of participants in terms of cadres, ages, clinical roles and areas of work (DHMT, patients, care providers). The deductive component of the analysis used a rigorous approach based on a combination of two acceptability frameworks, thereby expanding the aspects covered by our interview guide. Second, the combination of deductive and inductive approaches expanded the framework to touch on many more acceptability concepts and constructs, and capture further subjective, local impressions and ‘linguistic codes’, which contributed to the richness of the framework and later of the themes which are rooted in the local context.

The use of qualitative research means that we took a subjective stand; objectivity in this case is not really relevant or applicable. Our data were based on patients’ accounts of their personal views based on their experiences in the NCD clinics. Our approach does not allow for the generalization of these findings [61]. However, an in-depth understanding of the intervention’s acceptability has been provided through the diversified sample, descriptions of reported experiences and a combination of two data collection techniques (FGDs from patients and KIIs from patients, policy-makers and healthcare providers). Being a hypothetical study, the hypothetical product is not the same as the uptake of an actual product [62]; however, a hypothetical study is relatively inexpensive, simple for participants, and participants’ choices or selections are realistic [63]. As a pilot study, interpretation has to be made within the following limits: this is not a hypothesis-testing study, and does not necessarily allow for generalization beyond the inclusion and exclusion criteria [64]. However, despite these shortfalls, this approach fits well with the pilot stage of our research as the qualitative approach is very appropriate for problem identification, hypothesis generation, theory formation and concept development [65].

Focus groups and one-on-one interviews each present their own strengths and weaknesses, which can balance one another. FGDs may lead to some participants feeling uncomfortable in sharing their experiences in groups. Further, barriers and facilitators presented in the results may reflect those which were repeated in the FGDs, rather than those emerged from both the KIIs and FGDs [66].

Finally, as a theoretical framework was used, knowledge generated by this study is linked to work of others and the researcher’s experience. Also, the theoretical approach tends to reduce a complex intervention (BBCC + 5As + GS in our case) to key issues corresponding to the constructs of the framework used, rather than exploring the extent of experiences using a pure inductive approach [67].

Overall, the study provides many insights which can be used by policy makers and/or health care providers to successfully implement change in the realm of NCDs and increase the current acceptability level of BBCC + 5As +GS in Mangochi, Malawi. A future full trial could also use some of these findings during the design stage to increase its chance of success.

## Conclusion

BBCC + 5As + GS is acceptable and suggests a high likelihood of success for future use. However, caution must be exercised as several barriers may have to be overcome before successful implementation. Closely following suggestions given by participants for each of the themes and subthemes may result in an even more improved acceptability of BBCC in Mangochi. Based on these findings, it could be interesting to use a quasi-experimental design to see if this intervention would be feasible at the same site. Thus, future research should use the same study design, concurrently with a feasibility study, to see how well the intervention would work under controlled conditions (as an efficacy study).

## Data Availability

The datasets during and/or analysed during the current study are available from the corresponding author and can readily be accessed on reasonable request.

## Abbreviations

BBCC: Brief behaviour change counselling
5 A’s: Acronym of A (Ask), A(Advise/Alert), A(Assess), A(Assist) and A (Arrange)
MI: Motivational interviewing
GS: Guiding style from motivational interviewing
NCD: Noncommunicable diseases
LMICs: Low- and middle-income countries
DHMT: District health management team
KII: Key-informant interview
COMREC: College of Medicine Research and Ethics Committee
BSc.: Bachelor of Science
OPD: Outpatient department
ART: Antiretroviral therapy
DHO: District Health Office
CHAM: Christian Health Association of Malawi
GoM: Government of Malawi
HAS: Health surveillance assistant
IRB: Institutional Review Board
